# Reconstruction of gene innovation associated with major evolutionary transitions in the kingdom Fungi

**DOI:** 10.1186/s12915-022-01346-8

**Published:** 2022-06-15

**Authors:** Baojun Wu, Weilong Hao, Murray P. Cox

**Affiliations:** 1 School of Natural Sciences, Massey University, Palmerston North, 4410 New Zealand; 2 Department of Biological Sciences, Wayne State University, Detroit, MI USA

**Keywords:** Fungi, Gene gain, Horizontal transfer, Mitochondria, Trait transitions

## Abstract

**Background:**

Fungi exhibit astonishing diversity with multiple major phenotypic transitions over the kingdom’s evolutionary history. As part of this process, fungi developed hyphae, adapted to land environments (terrestrialization), and innovated their sexual structures. These changes also helped fungi establish ecological relationships with other organisms (animals and plants), but the genomic basis of these changes remains largely unknown.

**Results:**

By systematically analyzing 304 genomes from all major fungal groups, together with a broad range of eukaryotic outgroups, we have identified 188 novel orthogroups associated with major changes during the evolution of fungi. Functional annotations suggest that many of these orthogroups were involved in the formation of key trait innovations in extant fungi and are functionally connected. These innovations include components for cell wall formation, functioning of the spindle pole body, polarisome formation, hyphal growth, and mating group signaling. Innovation of mitochondria-localized proteins occurred widely during fungal transitions, indicating their previously unrecognized importance. We also find that prokaryote-derived horizontal gene transfer provided a small source of evolutionary novelty with such genes involved in key metabolic pathways.

**Conclusions:**

The overall picture is one of a relatively small number of novel genes appearing at major evolutionary transitions in the phylogeny of fungi, with most arising de novo and horizontal gene transfer providing only a small additional source of evolutionary novelty. Our findings contribute to an increasingly detailed portrait of the gene families that define fungal phyla and underpin core features of extant fungi.

**Supplementary Information:**

The online version contains supplementary material available at 10.1186/s12915-022-01346-8.

## Background

Fungi have existed for over a billion years and belong to the eukaryotic supergroup Opisthokonta, which comprises metazoans, choanoflagellates, filastereans, and ichthyosporeans [1]. Fungi inhabit an immense range of terrestrial and marine habitats and are highly diverse with up to 6.28 million species proposed to exist based on well-accepted non-parametric species estimators [2]. The most up-to-date taxonomy of the fungal kingdom comprises seven major groups with diverse genetics, morphologies, and life histories [3–5].

The Cryptomycota are thought to be the earliest diverging clade [6]. These intracellular parasites have a chitinous cell wall in their resting phase but not in their trophic phase [7, 8]. Members of the Cryptomycota have been found in fresh water, soil, sediment, and some marine habitats [9], indicating that the earliest diverging fungi were likely already adapted to both terrestrial and aquatic ecosystems. The next divergence leads to the phylum Blastocladiomycota and Chytridiomycota, which are free-living saprobes or parasitoids [10]. Some species in these two groups began to produce hyphae and pseudosepta (walls separating adjacent cells) [10]. Blastocladiomycota and Chytridiomycota are sister groups to the non-flagellated fungi (terrestrial fungi), although phylogenomic analyses have not resolved which group, Blastocladiomycota or Chytridiomycota, is closer to these non-flagellated fungi [5]. Non-flagellated fungi include the subkingdoms Zoopagomycota, Mucoromycota, and Dikarya (Basidiomycota and Ascomycota) [5]. The emergence of true hyphae, coupled with flagella loss, allowed this group of fungi to fully conquer land. Two other major changes occurred during this transition: (1) all stages of fungal life cycles from this evolutionary point on have true cell walls and (2) the spindle pole body acts as a microtubule-organizing center for mitotic and meiotic nuclear division [11]. Both changes helped expedite the long-distance dispersal of spores and resistance to adverse environmental conditions, compared to the earlier form of motile cells [12]. Land fungi evolved a new sexual structure, the zygospore, and thus the three non-flagellated fungi groups are also known as Zygomycetous fungi [10]. The earliest radiation of terrestrial fungi (Zoopagomycota) is associated with parasites/saprotrophs of animals, but terrestrial fungi gradually established extensive relationships with land plants, including as mycorrhizal fungi, root endophytes, and plant pathogens in the Mucoromycota and Dikarya [13, 14]. Mucoromycota seem to be the most ancient fungi that evolved to interact with plants as mycorrhizal fungi [13], receiving photosynthesis-derived carbon and providing the host plant with phosphorus and nitrogen in exchange. The Dikarya group has the largest number of described species and is the best-studied group of fungi today. They are characterized by a sexual cycle that includes heterokaryotic cells with unfused nuclei for a short (Ascomycota) or long (Basidiomycota) period after gamete fusion [3]. Although it is unclear why and how fungi maintain a dikaryotic stage prior to formation of a diploid nucleus, mating-type loci appear to control the dikaryon form [15, 16]. Compared with all other fungal phyla, Basidiomycota have yet another striking characteristic: they form the most complex multicellular structures among fungi, making sophisticated reproductive organs—fruiting bodies commonly known as mushrooms.

Identifying the genetic mechanisms associated with evolutionary transitions has recently gained increased attention thanks to rapidly growing genomic data. Several evolutionary transitions in fungi have been associated with gene loss and genomic duplications [17–20]. Emergence of novel genes across evolutionary transitions has been reported in animals and plants [21, 22], but is largely unknown in the third major group of eukaryotes, Fungi. To assess the role of novel genes at evolutionary transitions in fungi, we therefore apply a comparative genomics approach to systematically identify novel gene families and their functional repercussions at the points of major evolutionary fungal transitions.

## Results and Discussion

**Fig. 1:**
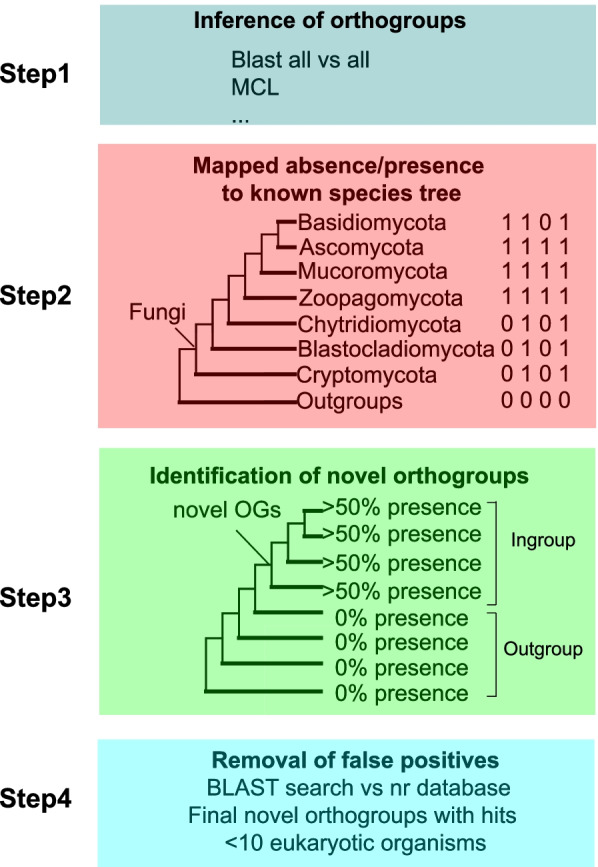
Summary of the analysis pipeline used to identify novel orthogroups

We used the genomes of 304 species across the phylogeny of fungi and other eukaryotes (see selection criteria in the Methods section) to interrogate the role of gene innovation at major evolutionary transitions in the kingdom Fungi. First, we identified homologous groups of proteins within and between species and determined their phylogenetic origin (see the Methods section and Fig. 1). We employed extensive taxon sampling and all-vs-all comparison of proteome homology instead of one-way BLAST (Step 1 in Fig. 1). Although commonly used, one-way BLAST is subject to homology detection failures, as emphasized by a recent study [23]. After a similarity search, homologs were clustered into orthogroups (OGs) using Markov modeling (Step 1 in Fig. 1). We compared orthogroups throughout the fungal species (ingroup) with their fungal or non-fungal outgroups through mapping the presence-absence pattern onto the known species tree (Steps 2 and 3 in Fig. 1). During this stage, we did not consider copy number variation, which is an important mechanism underlying fungal ecological transitions and has been widely studied [17, 18, 20, 24]. A novel orthogroup is defined as an orthogroup that is present in all phyla (at least 50% presence of all species in each phylum) of the clade being analyzed, and absent in all or all but one species in the outgroup (Step 3 in Fig. 1). Finally, proteins from the novel orthogroups were used for BLASTP searches against the NCBI non-redundant protein sequence database to exclude false positives since not all fungal genomes are sufficiently complete to be included in the main analysis (Step 4 in Fig. 1).

**Fig. 2:**
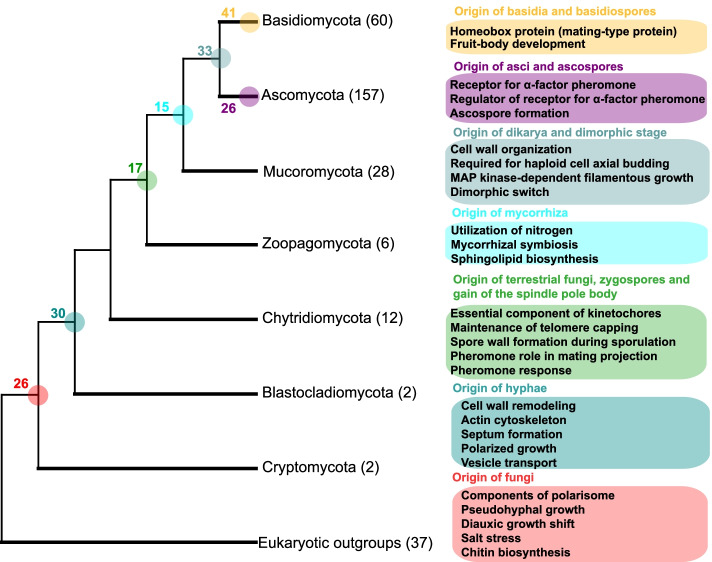
Biological functions of novel orthogroups based on *Saccharomyces cerevisiae* genes. Each colored box, coded by the phylogenetic group, is a summary of the extant biological processes that are associated with past evolutionary transitions. The number at each node represents the total number of novel orthogroups for that clade. Numbers in parentheses indicate the number of species studied in each phylum. The phylogenetic hypothesis of fungal phyla interrelationships is as per references in the text.

There are three caveats to the present study. First, the number of novel orthogroups identified here is very likely to be an underestimate because a 50% cutoff was used rather than the 10% cutoff employed in a previous study to identify novel orthogroups in the animal kingdom [25]. However, important orthogroups should logically be retained in a substantial number of species after their emergence, and a more conservative threshold was considered to be a better choice. In addition, a 50% cutoff is the minimum needed to allow a novel orthogroup to be present in at least one species in under-representative phyla, such as Cryptomycota and Blastocladiomycota (Fig. 2). Second, the number of species available from early diverging fungi is under-representative, possibly weakening the power to identify novel orthogroups. In particular, some novel orthogroups at very early stages of fungal evolution possibly may not meet the 50% cutoff without improved taxon sampling. To reduce this effect, our method requires that novel orthogroups are present in each phylogroup of the clade of interest after their first emergence (Step 3 in Fig. 1). This maximizes the probability of ancestral presence of novel orthogroups at the most recent common ancestor (MRCA) node. Third, proteomes in different phyla vary greatly, including in terms of protein number, orthogroup number and copy number within an orthogroup (Additional file 1: Fig. S1). Clades with more orthogroups would be expected to have more novel orthogroups, and vice versa. It is worth noting, however, that novel orthogroups are not only determined by ingroup size but also by their outgroup (Step 3 in Fig. 1). An obvious case is the clade of Mucoromycota and Dikarya, which have the second largest number of orthogroups (Additional file 1: Fig. S1), but exhibit the fewest novel orthogroups (Fig. 2). Therefore, the proteome size of phyla is not necessarily the key determinant for detecting novel orthogroups.

### The functions of novel orthogroups are associated with fungal evolutionary transitions

Using our analysis pipeline (see Fig. 1 for details), we identified 188 novel orthogroups across seven monophyletic super-phylum/phylum clades (Additional file 2: Table S1). The number of unique OGs in each clade ranges from 15 to 41 (Additional file 3: Table S2). In the paragraphs below, we elaborate on several instances where the origin of proteins and fungal transitions appear to coincide.

Twenty-six novel OGs occur at the most recent common ancestor (MRCA) of fungi, with functions in the polarisome, pseudo-hyphal growth, chitin biosynthesis, and diauxic growth shift (Fig. 2 and Additional file 3: Table S2). These traits are typical of fungal lineages [26] and the polarisome seems to distinguish fungi from other eukaryotic clades [27]. One novel OG against salt stress (NHA1) arose in the earliest diverging fungi. This OG is likely essential for survival under high salinity and is consistent with a proposed marine origin of fungi [28]. In addition, two important signaling pathways, including MAPK and TOR, innovated at the origin of fungi (Additional file 4: Table S3).

We identified 30 novel OGs at the MRCA of Blastocladiomycota and its sister phyla, among which are genes associated with the origin of hyphae. This includes novel OGs encoding proteins for cell wall remodeling, actin cytoskeleton and septum formation, polarized cell growth, and vesicle transport, all of which are related to hyphae morphogenesis (Fig. 2 and Additional file 3: Table S2) [17].

Zoopagomycota is the most basal lineage of the zygomycetous fungi and is associated with three major phenotypic transitions in the history of fungal evolution: (1) the origin of full terrestrialization, (2) the emergence of zygospores, and (3) gain of the spindle pole body (SPB). Novel OGs were found for all those changes. The transition to terrestrial environments is facilitated by mitotic and meiotic spore formation and resistance to adverse environmental conditions. Two OGs encode proteins of telomere capping and spore wall formation (Fig. 2 and Additional file 3: Table S2), both of which are known to improve resistance to adverse environments. Two other OGs have pheromone-related functions, which are associated with the emergence of zygospores (Fig. 2 and Additional file 3: Table S2). Unexpectedly, no novel OGs are directly linked to the appearance of the SPB (Additional file 1: Fig. S2). However, three novel OGs (DAD4, ASK1, and DUO1) encode essential components of kinetochores, which play important roles in the function of the spindle pole body (SPB) [29]. Another three novel OGs (DAM1, DAD3, and KAR9), which encode proteins with SPB-related functions, were also found at the MRCA of Mucoromycota and its sister phyla, suggesting that the development of the function of the spindle pole body was a successive process and also noting the importance of mitotic and meiotic spores. Mucoromycota is the second most basal group of terrestrial fungi with a wide range of interactions with land plant hosts [13, 30]. Consistent with this lifestyle, we found that one novel OG (LUG1) is associated with plant symbiosis [31], and another novel OG (SHR3) is associated with the utilization of nitrogen [32] (Fig. 2 and Additional file 3: Table S2). One OG involved in sphingolipid biosynthesis was also found. Previous studies have suggested important roles for lipids in fungi-plant interactions [33, 34].

Dikarya is by far the most well described fungal superclade, and 33 novel OGs were found at its origin point. Consistent with the view that the origin of dimorphism coincided with the appearance of dikaryons [35], a novel OG (UME6) plays a key role as a dimorphic switch [36, 37]. In addition, proteins encoding functions of cell wall assembly regulation, haploid cell axial budding, and filamentous growth were also found. Basidiomycota and Ascomycota are the two major phyla within Dikarya. Although both phyla have dikaryotic growth, the dikaryotic stage is more significant in Basidiomycota than in Ascomycota [11]. Ascomycota have two novel OGs (STE2 and LDB19) that encode a receptor for the mating hormone alpha-factor and its regulator, while Basidiomycota have a novel OG that encodes a mating-type protein (Fig. 2 and Additional file 3: Table S2). These novel OGs have been linked to the development of the dikaryon [15, 16, 38], suggesting that dikaryotic formation may have evolved independently in these two groups. Basidiomycota and Ascomycota have characteristic spore-producing cells, basidia in fruiting bodies and asci in ascomata, respectively. The corresponding spores produced are called basidiospores and ascospores, respectively. We identified two novel OGs, including four proteins (RIM21, FMP45, YNL194C, and SUR7), that contribute to ascospore formation in Ascomycota (Fig. 2, Additional file 3: Table S2 and Additional file 4: Table S3). In Basidiomycota, few annotations are available to explain the associations between traits and novel OGs. However, 20 of the 41 novel OGs (49%) are involved in fruit-body development based on a gene expression analysis in *Rickenella mellea* [39].

From these results, it appears that a substantial fraction of the identified novel OGs are associated with major fungal transitions (Fig. 2 and Additional file 3: Table S2 and Additional file 4: S3), although we note that correlations cannot prove a causal relationship in the absence of ancestral genetic information. The other novel OGs identified in our study do not show clear phenotypic characteristics specific to any one fungal group (Additional file 3: Table S2). For instance, Gene Ontology (GO) terms for cellular copper ion homeostasis (MRCA of Blastocladiomycota and other phyla) and positive regulation of glycerol transport (MRCA of fungi) are enriched among the novel OGs (Additional file 4: Table S3). It is worth noting that annotations are retrieved from *Saccharomyces cerevisiae* homologs, but the same OG may have different functions in other species.

### Mitochondria-localized novel proteins widely occur at transition nodes

**Fig. 3:**
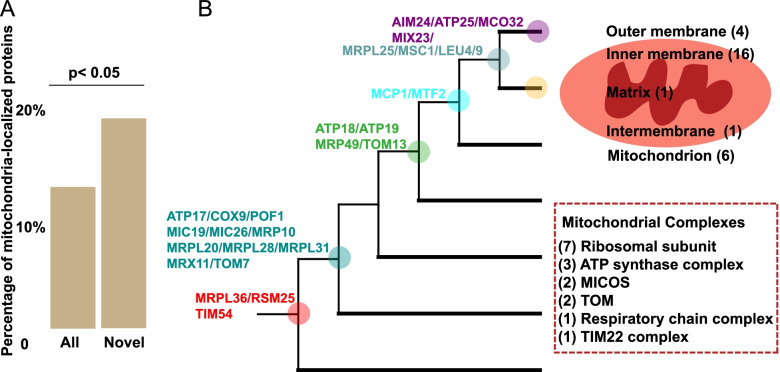
Mitochondria-localized novel orthogroups. **A** The percentage of mitochondria-localized proteins among novel proteins (Novel) is significantly higher than those among all proteins (All). **B** Proteins mapped on the phylogeny represent mitochondria-localized novel proteins identified by the analysis pipeline in Fig. 1. The cartoon in the upper right panel shows subcellular location and the dashed rectangle in the lower right panel shows proteins involved in major mitochondrial complexes

The 188 novel OGs that we identify have 139 homologs in the model organism *Saccharomyces cerevisiae*. We checked the proteins listed as localizing at mitochondria with the literature [40] and found 28 of 139 proteins (20%) are robustly annotated (Additional file 5: Table S4). It is worth noting that the mitochondria-localized proteins discussed here are encoded by the nuclear genome, not the mitochondrial genome. We tested whether mitochondria-localized proteins tend to be novel orthogroups compared with a random background distribution of 882 high-confidence mitochondria-localized proteins [40] of the total 6681 nuclear proteins in *Saccharomyces cerevisiae*. Mitochondria-localized proteins are significantly enriched among novel OGs (Fig. 3A, Fisher’s test, two-tailed, *p*<0.05), but the significance may vary in other species. Unfortunately, there is no information on protein subcellular location in fungi other than *S. cerevisiae*. Mitochondria-localized novel proteins are found at all major transitions except that leading to Basidiomycota (Fig. 3B). *S. cerevisiae* gained another 13 species-specific mitochondria-localized proteins compared to other fungi (Additional file 5: Table S4), thus suggesting that mitochondria-localized proteins remain a persistent innovation in fungal evolution. Submitochondrial location analysis reveals that more than half of mitochondria-localized proteins (16 proteins) occur at the inner membrane, followed by an unknown submitochondrial localization (6 proteins), then location in the outer membrane (4 proteins) (Fig. 3B). Of the 28 mitochondria-localized proteins, 16 (57%) are components of key mitochondrial complexes, such as the mitochondrial ribosomal complex and mitochondrial cristae complex (Fig. 3B). Interestingly, a recent study also identified mitochondrial ribosomal proteins and mitochondrial contact site and cristae organizing system (MICOS) proteins as bilaterian-specific gene gains compared with other eukaryotic organisms [25].

Previous studies have implied that the complex evolution of mitochondria-localized proteins involved numerous gain and loss events in fungi and other eukaryotes [41, 42]. Here, we found that gain of mitochondria-localized proteins occurred at six MRCA nodes (Fig. 3). Potentially, the gain of mitochondria-localized proteins that associate with modern fungal mitochondrial proteomics might reflect specific adaptations to mitochondria-specific functions. As evidence for adaptation, 11 (39%) of these mitochondrial associated genes arose at the MRCA of Blastocladiomycota and sister groups, which corresponds to the lifestyle transition from parasitoids to saprobes (Fig. 3B and Additional file 1: Fig. S3). GO enrichment analysis further reveals cristae formation (GO:0042407) at this node (Additional file 4: Table S3), which is the main site for oxidative phosphorylation and important for cellular energy production [43]. In addition, given the important role of fungal mitochondria in virulence, pathogenicity, drug resistance, and metabolism [44], the increased complexity of mitochondria themselves may contribute to fungal evolution. Mitochondria-localized proteins can have functions well beyond the mitochondria. Recent studies suggest that mitochondrial ribosomal proteins play important roles in development in both plants and animals [45–47]. However, it is unclear whether they play similar roles across the Fungi kingdom, and further analyses will be needed to shed more light on this matter.

### Novel OGs are functionally connected

**Fig. 4:**
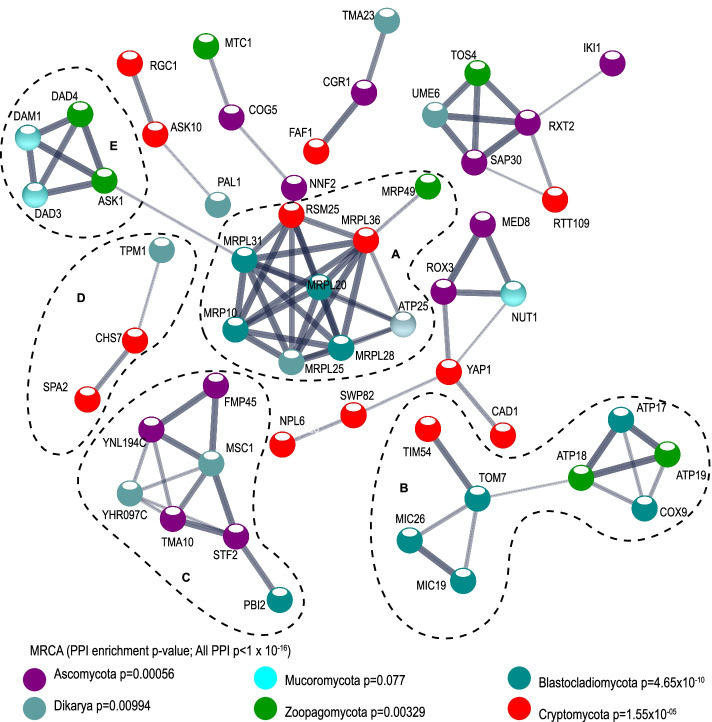
Protein-protein interaction network of novel proteins. Protein names corresponding to the *S. cerevisiae* equivalents of 139 novel proteins were uploaded to the STRING database. Only networks including at least three proteins are shown

We analyzed potential interactions among novel OGs using the STRING protein-protein interaction (PPI) database and the corresponding *Saccharomyces cerevisiae* homologs. The network of all novel proteins has significantly more interactions than expected (PPI enrichment, *p*<1.0×10^−16^), revealing that novel genes form extensive networks where ~46% of the proteins (65/139 proteins) are connected to each other (Fig. 4). We also checked PPI at each MRCA node and found that all but one have significantly more interactions than expected (Fig. 4). The exception is the MRCA of Mucoromycota and sister groups, which is the node with the fewest novel OGs. These observed connections indicate that the proteins are at least partly biologically connected as a group. Among these networks, mitochondria-localized novel proteins are included in three large networks (networks A, B, and C in Fig. 4). These interactions also form several other networks involved in important aspects of fungal evolution, such as cell wall regulation (network D) and the function of the spindle pole body (network E). For each network, the proteins have different origins (different colors in Fig. 4) suggesting that these innovations have evolved in an incremental fashion.

### Prokaryote-derived novel orthogroups shape mosaic biosynthesis pathways

We employed an approach that combines an all-vs-all BLAST search with clustering. If a new gene arises from a duplication event, then both parental and child copies would be clustered into the same orthogroup in the relevant clade and the orthogroup would be shared by the species in the outgroup. Therefore, the novel orthogroups identified in this study mostly did not result from duplications but instead most likely derive from either de novo formation or horizontal gene transfer (HGT). We found that two novel OGs (1% of the total) arose from bacteria through HGT. The number of novel orthogroup genes that arose through de novo formation thus far outnumber those derived by prokaryote-derived HGT.

**Fig. 5:**
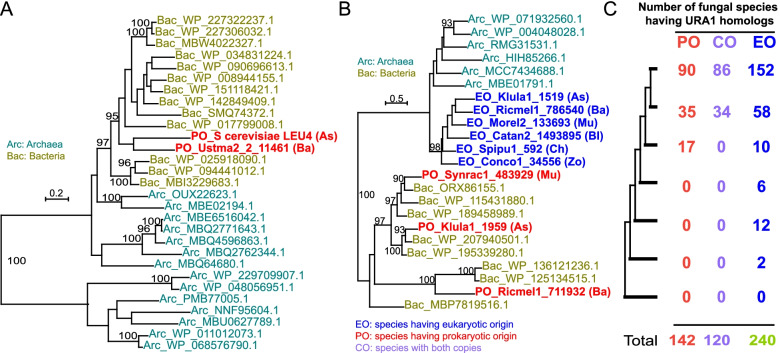
Prokaryote-derived horizontal gene transfers. **A** Phylogenetic relationship of LEU4 from selected species using IQ-TREE2. Only bootstrap values >90% are shown. As, Ascomycota; Ba, Basidiomycota. **B** Phylogenetic relationship of dihydroorotate dehydrogenases from selected species using IQ-TREE2. Mu, Mucoromycota; Zo, Zoopagomycota; Ch, Chytridiomycota; Bl, Blastocladiomycota. **C** Distribution of dihydroorotate dehydrogenase in fungi identified by our pipeline (see Additional file 6: Table S5)

The biosynthesis of leucine is a common pathway in prokaryotes, plants, and fungi, but absent from animals, including humans. LEU4 (a mitochondria-localized protein) encodes an alpha-isopropylmalate synthase that catalyzes the first step in leucine biosynthesis. Our results indicate that HGT-derived LEU4, as a novel OG, contributed to leucine biosynthesis in Dikarya (Fig. 5A) and this enzyme can allow its hosts to consume additional substrates. Our result shows that the LEU4 protein is not present in other fungal species beyond dikarya and eukaryotic outgroups. If the LEU4 pattern resulted from mitochondria-to-nuclear transfer followed by loss, these results would require a massive number of independent events, which is therefore considered highly unlikely. Instead, one prokaryote-derived transfer is the more parsimonious explanation for the pattern. In addition, analyses of mitochondrial genomes in fungi also discount the pattern arising from mitochondrial to nuclear genome transfer.

The fourth enzyme in the de novo biosynthesis of pyrimidines (dihydroorotate dehydrogenase, DHODH), known as URA1 in fungi, converts dihydroorotate to orotate [48]. We found that dihydroorotate dehydrogenases (DHODHs) are separated into two distinct orthogroups in fungi (OG0002720 and OG0005207, Additional file 1: Fig. S4). Some fungal species, such as *Kluyveromyces lactis*, carry gene copies from both orthogroups (Additional file 6: Table S5). In *K. lactis,* the two copies of DHODHs share only 24% sequence identity with each other, suggesting a different origin. Leveraging the *K. lactis* protein sequences, we investigated the origin of each orthogroup through BLAST searches against the NCBI nr database outside the fungal kingdom. OG0005207 (Klula1_1959) has a prokaryotic origin, whereas OG0002720 (Klula1_1519) has a eukaryotic origin. To confirm the BLASTP results, random DHODHs from different phyla were used for small-scale phylogenetic inference. The DHODHs with a prokaryotic origin clustered with bacterial homologs rather than homologs with eukaryotic origins (Fig. 5B). The tree pattern also indicates that horizontal gene transfer events possibly occurred multiple times to shape the modern pattern of URA1 (Fig. 5B). Systematic examination of 267 fungal species shows that the prokaryotic copy is present in Mucoromycota and sister groups, and both prokaryotic and eukaryotic copies co-exist in most sequenced Dikarya genomes (Fig. 5C and Additional file 6: Table S5). Since most animals have the dihydroorotate dehydrogenase gene, the absence of the eukaryotic copy of DHODHs in Cryptomycota suggests independent gene loss, which is also evident in some individual Dikarya species, such as *Blastomyces dermatitidis* and *Moesziomyces aphidis* (Additional file 6: Table S5), where both copies have been lost. The prokaryotic copy of URA1 has replaced the eukaryotic copy in 22 species, mostly in the phylum of Mucoromycota (Fig. 5C and Additional file 6: Table S5). The replacement of the eukaryotic gene by the horizontally-acquired prokaryotic gene can have significant functional consequences. For instance, *Saccharomyces cerevisiae* contains only the horizontally-acquired prokaryotic copy of URA1, which has been proposed to facilitate improved growth under anaerobic conditions than the eukaryotic copy of URA1 [49]. It is possible that the origin of the prokaryotic copy is associated with the mycorrhizal revolution in the phyla of Mucoromycota and its sister groups.

## Methods

### Genomic datasets

A detailed description of the pipeline developed and used here can be found in Fig. 1. Briefly, proteins taken from whole genome sequences were used to identify orthogroups (OGs) within and between phyla. Broad taxonomic sampling of genomic data was implemented to accurately infer the phylogenetic origin of different OGs. 267 fungal genomes (seven phyla and 25 subphyla) and 37 genomes from a diverse representation of eukaryotic outgroups were studied (Additional file 7: Table S6). BUSCO 3.1.0 [50] was used to assess the quality of the genome annotations. Three criteria were used to select the fungal genomes: (1) only published genomes were included for replicability and use-permissions reasons; (2) genomes had to have a BUSCO completeness >90%; and (3) two genomes (if available) were selected from each fungal family, with one being relatively early-branching and the other being a late-branching species (based on the JGI phylogenetic tree) to better represent genomic diversity.

### Orthogroup assignment

Sequence similarity for all predicted proteins was identified with an all-versus-all diamond BLASTP 2.0.4 search using an *E*-value of 10×10^−5^, *k*=100 and the “very sensitive” model. This model is designed to find distant hits of <40% identity with a sensitivity similar to BLASTP [51]. Higher inflation values during the Markov Cluster Algorithm (MCL) clustering can result in higher numbers of orthogroups (that is, an orthogroup that has undergone a high number of duplications will be split into several smaller orthogroups). We therefore chose an inflation value of 1.5 as a conservative approach in MCL 14–137 [52] as discussed in other studies [52, 53]. All steps were performed using Orthofinder 2.3.14 [54], which has the additional property that gene length bias can be accounted for in orthogroup detection.

### Identification of novel orthogroups

There has been no prior systematic study to identify novel orthogroups in the fungal kingdom. For the animal kingdom, previous studies have either employed at least 10% or 95% presence to identify phylum-specific orthogroups [25, 55]. The lower cutoff does not capture the relative importance of orthogroups (important orthogroups should logically be retained in a substantial number of species), whereas the higher cutoff ignores the extensive gene loss that is observed during evolution. To balance these factors, we required a novel OG to be present in at least 50% of species after its point of emergence. For the “terminal” Ascomycota and Basidiomycota phyla, we required that a protein must be present in at least 50% of the species in each sub-phylum. In short, a novel OG must be present in 50% of lineages within a clade and also be absent in taxa outside the clade of interest (or present only once to allow for some level of HGT or database errors). As discussed in a previous study [22], the likelihood of false positives and negatives is reduced because each OG generally contains multiple genes per genome.

### Novel orthogroup validation

To confirm accurate identification of novel orthogroups, *Saccharomyces cerevisiae* proteins and proteins from species in the early divergent phylum/subphylum for each OG were tested by performing BLASTP searches against the NCBI nr database (last accessed 03/2022) excluding the clade of interest. If novel OGs were not present in *S. cerevisiae*, those from *Neurospora crassa* in the Ascomycota were used as alternatives. Because Basidiomycota-specific novelties do not have homologs in other fungi, including *S. cerevisiae* (Ascomycota), *Ustilago maydis* proteins from Ustilaginomycotina and *Leucosporidiella creatinivora* proteins from Pucciniomycotina were used for the BLASTP validation. Both subphyla are early diverging subphyla within the Basidiomycota. If a candidate OG had <10 non-fungi eukaryotic organisms with homologs, it was regarded that the OG arose as a novel OG. This approach allowed the maximum breadth of taxonomic sampling to minimize false positives (Fig. 1).

### Functional annotations and PPI network analyses

Alignments were generated by mafft 7.215 [56] and trimmed by trimAI 1.4 with the automated1 option [57]. The two phylogenies in Fig. 5A, B were constructed with IQ-TREE 2 [58] with the best model determined according to BIC and 1000 ultrafast bootstrap replicates. The tree in Additional file 1: Fig. S4 was constructed with fasttree 2 with the -lg -gamma -spr 4 -mlacc 2 -slownni options [59]. To obtain functional descriptions for novel OGs, their *Saccharomyces cerevisiae* homologs were assessed. Subcellular protein locations were obtained from Uniprot [60] and published literature [40]. Protein interaction data were obtained from the STRING 11.5 database of known and predicted protein-protein interactions [61]. To construct PPI networks, we uploaded *Saccharomyces cerevisiae* IDs to the STRING browser interface. Parameters for the displayed PPI network were: three interaction sources (curated databases, experimentally determined, and co-expression); a minimum required interaction score of 0.4; and a maximum number of interactors to display in the first and second shell set to zero. SGD gene identifiers (IDs) of novel proteins were downloaded from the SGD database and then submitted to DAVID 6.8 [62] to perform GO enrichment analysis, with a default EASE cutoff of 0.1.

## Supplementary Information

**Additional file 1: Figure S1.** Protein number (A), orthogroup number (B), core orthogroup number (C) and copy number (D). Protein number and orthogroup number are shown for each phylum; core orthogroup number refers to orthogroups that are present in at least 50% of species after their emergence; copy number is the protein number in each core orthogroup. **Figure S2.** Distribution of proteins involved in formation of the spindle pole body. Black boxes represent “presence”; gray boxes represent “absence”. **Figure S3.** Distribution of proteins involved in three mitochondrial ultrastructure complexes. Black boxes represent “presence”; gray boxes represent “absence”. **Figure S4.** Maximum likelihood tree of 382 fungal dihydroorotate dehydrogenases (142 prokaryotic origin and 240 eukaryotic origin). The tree is midpoint rooted. Klula1_1519 and Klula1_1959 were used to determine the origin of each orthogroup. The best hit and frequency of organisms among the top 100 best hits are also shown.

**Additional file 2: Table S1.** 188 novel orthogroups among 267 fungal species.

**Additional file 3: Table S2.** Annotation of 188 novel orthogroups.

**Additional file 4: Table S3.** GO enrichment analysis of novel orthogroups based on *Saccharomyces cerevisiae* homologs.

**Additional file 5: Table S4.** Details of mitochondria-localized novel proteins.

**Additional file 6: Table S5.** Distribution of dihydroorotate dehydrogenases among 267 fungal species.

**Additional file 7: Table S6.** Genomes used in this study, species code, BUSCO scores and phylum information.

## Data Availability

All genomic data used in this manuscript are publicly available. Access details are listed in Additional file 7: Table S6.

## Abbreviations

BIC: Bayesian information criterion
DHODH: Dihydroorotate dehydrogenase
GO: Gene ontology
HGT: Horizontal gene transfer
MCL: Markov cluster algorithm
MICOS: Mitochondrial contact site and cristae organizing system
MRCA: Most recent common ancestor
OG: Orthogroup
PPI: Protein-protein interaction
SGD: Saccharomyces genome database
