# Supplementary material for: Reconstruction of gene innovation associated with major evolutionary transitions in the kingdom Fungi
Source: BMC Biol. 2022 Jun 15;20:144. doi: 10.1186/s12915-022-01346-8 (PMC9202105; doi:10.1186/s12915-022-01346-8)
Supplement: Supplementary file 1 — Additional file 1: Figure S1. Protein number (A), orthogroup number (B), core orthogroup number (C) and copy number (D). Protein number and orthogroup number are shown for each phylum; core orthogroup number refers to orthogroups that are present in at least 50% of species after their emergence; copy number is the protein number in each core orthogroup. Figure S2. Distribution of proteins involved in formation of the spindle pole body. Black boxes represent “presence”; gray boxes represent “absence”. Figure S3. Distribution of proteins involved in three mitochondrial ultrastructure complexes. Black boxes represent “presence”; gray boxes represent “absence”. Figure S4. Maximum likelihood tree of 382 fungal dihydroorotate dehydrogenases (142 prokaryotic origin and 240 eukaryotic origin). The tree is midpoint rooted. Klula1_1519 and Klula1_1959 were used to determine the origin of each orthogroup. The best hit and frequency of organisms among the top 100 best hits are also shown. [file 12915_2022_1346_MOESM1_ESM.pdf]

Reconstruction of gene innovation associated with major evolutionary transitions in the kingdom Fungi

Baojun Wu[1*], Weilong Hao[2], Murray P. Cox[1*]

[1] School of Natural Sciences, Massey University, Palmerston North 4410, New Zealand

[2] Department of Biological Sciences, Wayne State University, Detroit, MI, USA

[*] Corresponding Authors:

Murray Cox, School of Natural Sciences, Massey University, Palmerston North 4410, New Zealand.

Email: m.p.cox@massey.ac.nz; Phone: +64 6 951 7747

ORCID ID: orcid.org/0000-0003-1936-0236

Baojun Wu, School of Natural Sciences, Massey University, Palmerston North 4410, New Zealand.

baojun.edison.wu@gmail.com

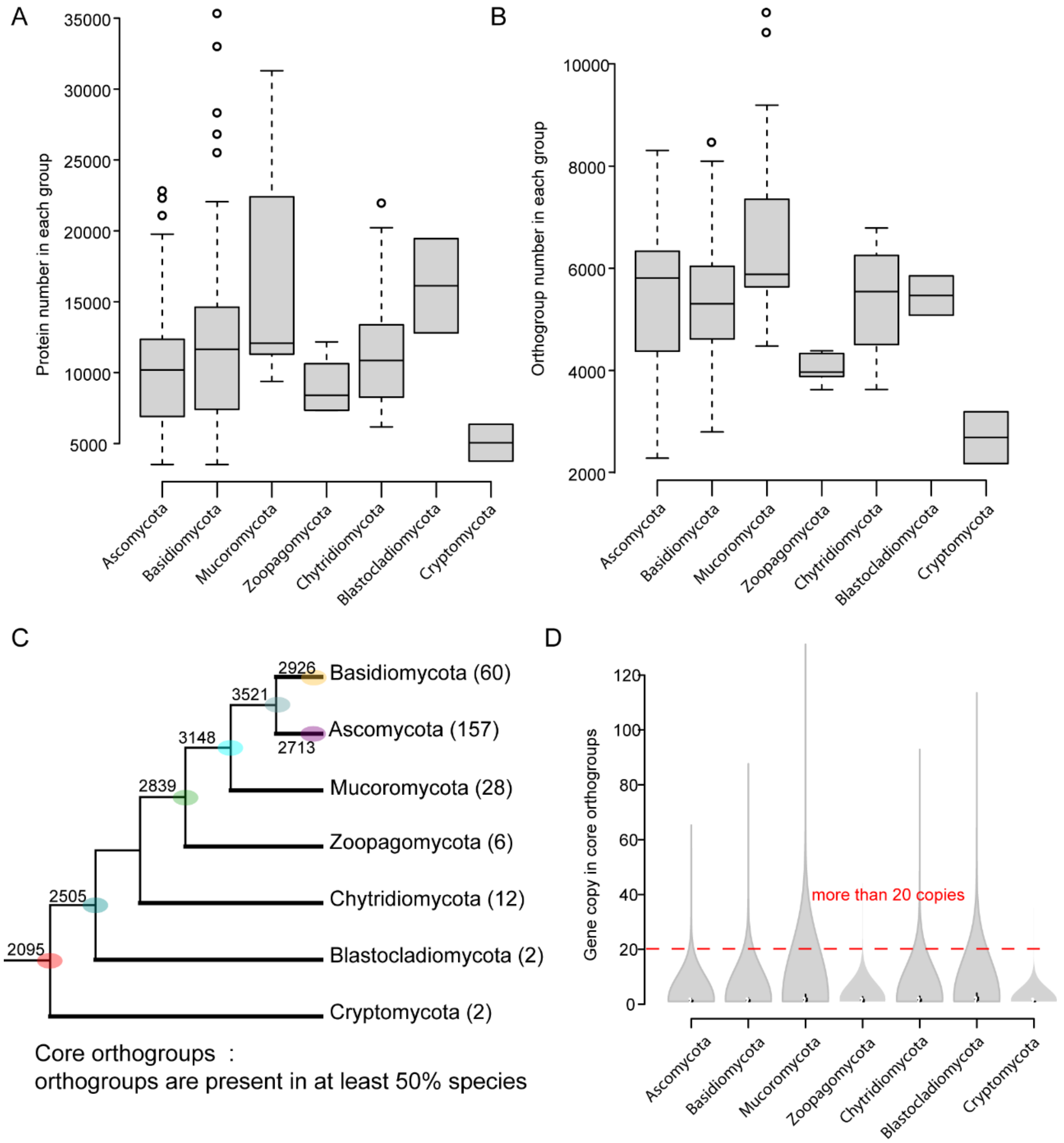

Figure S1. Protein number (A), orthogroup number (B), core orthogroup number (C) and copy number (D). Protein number and orthogroup number are shown for each phylum; core orthogroup number refers to orthogroups that are present in at least 50% of species after their emergence; copy number is the protein number in each core orthogroup.

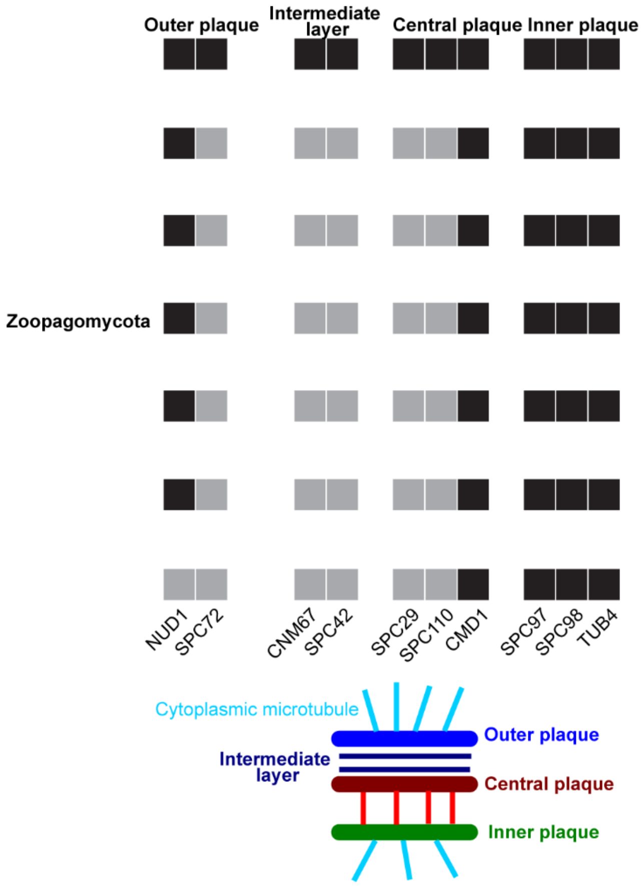

Figure S2. Distribution of proteins involved in formation of the spindle pole body. Black boxes represent "presence"; gray boxes represent "absence".

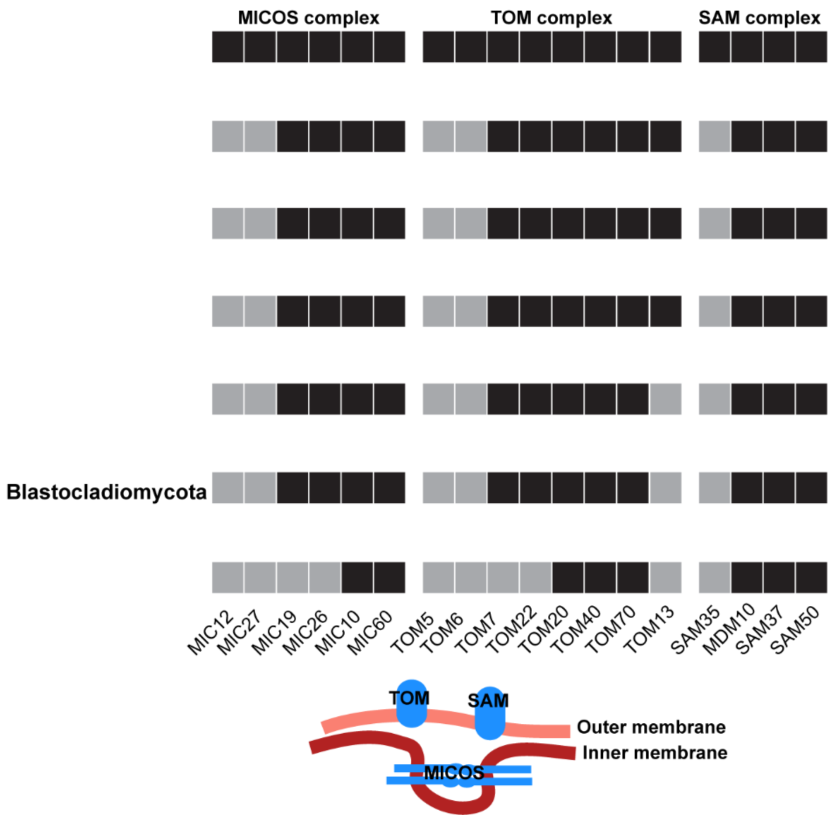

Figure S3. Distribution of proteins involved in three mitochondrial ultrastructure complexes. Black boxes represent “presence”; gray boxes represent “absence”.

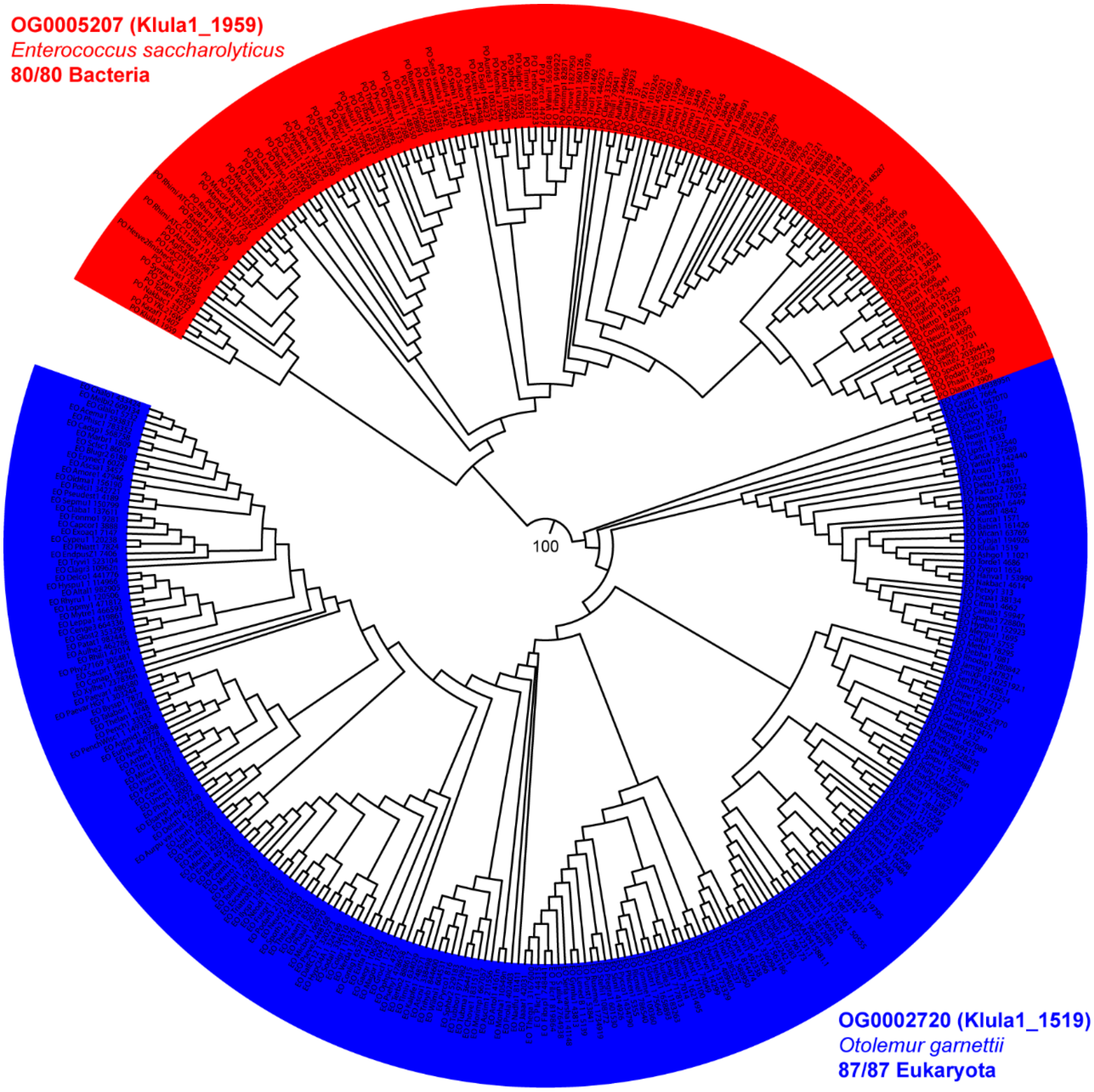

Figure S4. Maximum likelihood tree of 382 fungal dihydroorotate dehydrogenases (142 prokaryotic origin and 240 eukaryotic origin). The tree is midpoint rooted. Klula1_1519 and Klula1_1959 were used to determine the origin of each orthogroup. The best hit and frequency of organisms among the top 100 best hits are also shown.
